# From Chromosomal Aberrations to Transcriptome Analysis: Four Decades of Research in Bivalve Genotoxicity

**DOI:** 10.3390/ijms26115389

**Published:** 2025-06-04

**Authors:** Zenaba Khatir, Alexandra Leitão

**Affiliations:** 1 Environmental Science Center, Qatar University, Doha 2713, Qatar; zenaba.khatir@qu.edu.qa; 2 LEMAR, University of Brest/CNRS/Ifremer/IRD, F-29280 Plouzané, France

**Keywords:** genotoxicity, bivalves, cytogenetics, DNA, gene, transcriptomics, molecular responses, chemical stressor

## Abstract

Over the past four decades, bivalves have become sentinel organisms in genotoxicity research due to their ecological relevance and sensitivity to environmental contaminants. This integrative review critically examines the evolution of genotoxicity in bivalves, from early cytogenetic assays to advanced transcriptomic approaches. It highlights key methodological developments, geographical research trends, and the recent integration of multi-endpoint analyses for a more robust, consistent environmental risk assessment. By synthesizing data from four decades of research, we provide a comprehensive overview of current knowledge while also critically identifying persistent challenges and suggesting directions for future research to allow better evaluation and mitigation of the genetic impacts of marine pollution.

## 1. Introduction

Organisms inhabiting coastal areas are exposed to a wide range of physical and chemical stressors originating from human activities. These stressors, which include heavy metals, pesticides and industrial chemicals, physical pollutants such as microplastics, or altered sediment dynamics, among other factors, leach into the marine environment, causing adverse effects on marine life [1,2,3]. Despite past and ongoing research, there remains a lack of comprehensive understanding of their full impact on marine ecosystems [1]. One main concern is the ingestion and bioaccumulation of chemical substances that are prevalent in the marine environment by marine organisms [4,5,6]. Some of these widespread substances can induce alterations and changes in genetic material at the DNA or chromosomal level; hence, they are called genotoxins [7,8]. The Globally Harmonized System of Classification and Labeling of Chemicals (GHS) has defined genotoxins and genotoxicity as “agents or processes which alter the structure, information content, or segregation of DNA, including those which cause DNA damage by interfering with normal replication processes, or which in a non-physiological manner (temporarily) alter its replication” [9]. Genotoxins can initiate a cascading, delayed effect, beginning at low biological levels and causing modifications in the genetic material even at non-lethal, non-cytotoxic concentrations. These alterations often result in delayed consequences at the cellular level, which can extend to the organism and potentially lead to prolonged impacts at the population and community levels [10,11].

To fully understand and mitigate these potential risks, it is essential to employ tools with a wide application range that extends beyond the scope of classic toxicology. The growing concern over the increasing bioavailability of (geno)toxic agents in the marine environment has driven the development of more sophisticated approaches/advanced methodologies that better assess their biological impacts. This has led to the establishment of a new specialized field of toxicology—genetic toxicology, also known as genotoxicology or genotoxicity—dedicated to investigating the carcinogenicity and mutagenicity of these compounds at the genetic level [8,10].

**Figure 1:**
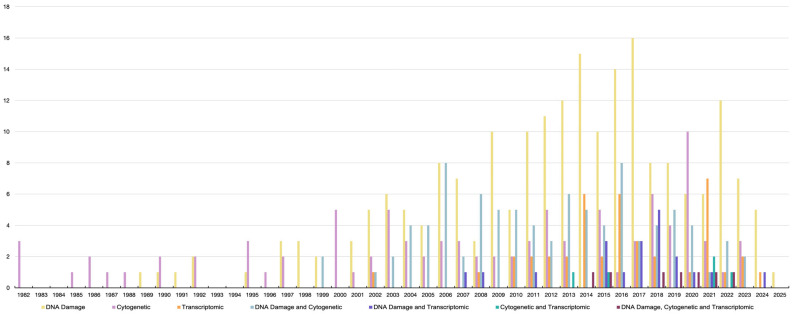
Published genotoxic data by endpoint in chronological order from 1982 to 2025.

**Figure 2:**
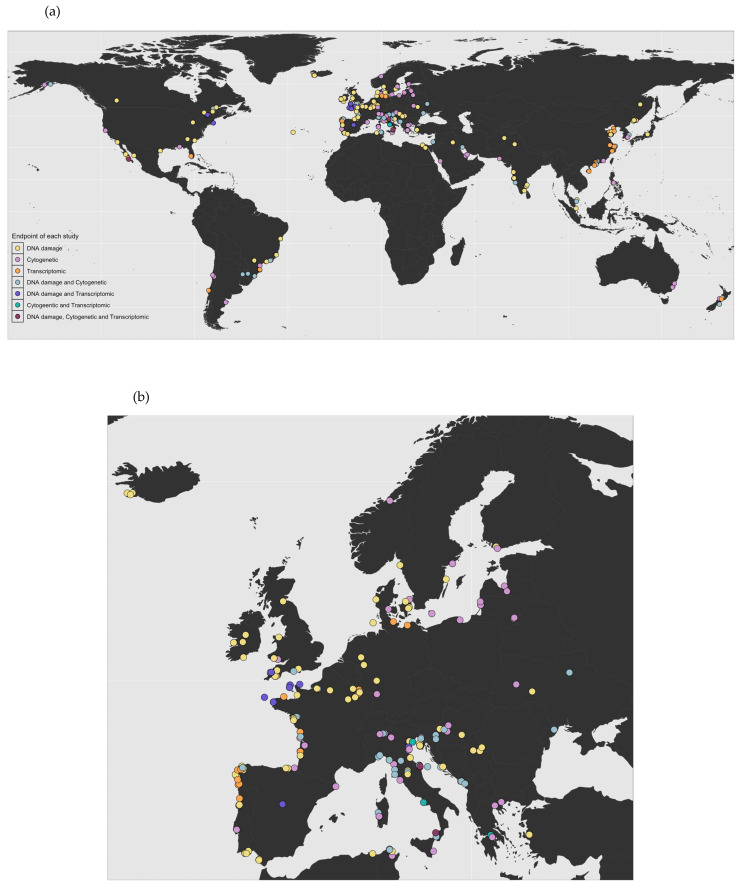
Geographical distribution of published genotoxicity studies by endpoint: (**a**) worldwide; (**b**) Europe (R package version 1.0.0.9000. 2025).

In the early 1980s, the first reports on genotoxicity testing in bivalves were published (Figure 1). Since then, bivalves have become focal organisms in genotoxicity research, mainly due to their wide ecological distribution, sessile lifestyle, feeding mechanisms, and relatively short maturation cycle. Initial studies on bivalve genotoxicity employed experimental and monitoring approaches to examine a wide range of stressors. During the first decade of genotoxicity research, studies on bivalves were limited to Italy, the UK, and Croatia. Out of 470 studies performed between 1982 and 2025, 313 were carried out in Europe, with Italy taking the lead with 77 studies, followed by the UK and France with 55 and 47 studies, respectively (Figure 2a,b). Since 2004, there has been a significant increase in bivalve genotoxicity research in China, with 35 studies published and around half of them using omics endpoints (Figure 2a). Species-wise, the majority of studies carried out so far have used *Mytilus* sp. as the model species, representing 45% of the studies. However, the number of model species has been steadily increasing, with several other species currently being used in genotoxicity assays, such as *Crassostrea* sp., *Dreissena* sp., and *Anodonta* sp.

This integrative review aims to provide a comprehensive summary of the published data on genotoxicity assays applied to bivalve tissue and analyze the different methodologies used to assess environmental genotoxicity in marine bivalves, examining their evolution over time and across geographic regions. Major scientific databases (including Web of Knowledge (Web of Science), Scopus, PubMed, Springer Link, and Google Scholar) were searched, primarily using a combination of the following keywords: “Genotoxic”, “bivalve”, “oyster”, “mussel”, “clam”, “cockle”, “scallop”, “contaminant”, and “pollution”. Studies were excluded if they did not involve genotoxicity investigations, did not utilize a bivalve model, or employed assays applied exclusively to mammalian cell lines or bacterial systems rather than directly to bivalve tissues. By synthesizing the results of the published studies, this review offers a clearer picture of the impact of environmental genotoxins on marine bivalves. This critical analysis of the research published so far aims to support future studies and contribute to the development of more effective strategies for monitoring and mitigating the adverse effects of chemical pollutants on marine life.

## 2. Endpoint Evolution over Time and Across Geographic Regions

### 2.1. Cytogenetic Endpoints

The earliest reports on bivalve genotoxicity focused on utilizing chromosome-level endpoints, such as chromosomal aberrations—both structural and numerical (aneuploidy)—and sister chromatid exchange (SCE) ([12,13,14]; Figure 1). These studies provided crucial information on the non-reversible impact on chromosomes that can be observed during cell division. Both aneuploidy and SCE levels displayed high sensitivity in detecting the genetic impact of elevated concentrations of heavy metals and hydrocarbons in mussel tissue (Supplementary Table S1). A consistent dose–response relationship between these endpoints and different toxicants was repeatedly observed, highlighting their importance in early environmental monitoring efforts.

During the first decade of genotoxicity studies on bivalves, two-thirds of the publications focused on cytogenetics and numerical chromosomal aberrations in particular, mainly in mussels and specifically *Mytilus* spp. (Figure 1). These direct chromosomal aberration and SCE tests require the capture of dividing cells in specific phases of the cell cycle.

In contrast, the micronucleus assay does not rely on cell division staging. Micronuclei can be observed in interphase cells and are formed by DNA fragmentation after stress, exposure, or chromosome mis-segregation during cell division. In the last case, a whole chromosome or part of it lags behind in anaphase, condenses, and then proceeds to the next division cycle independently of the main nucleus. This assay rose in popularity because it is a simple and inexpensive method with high sensitivity. From 1987 to 2025, the micronucleus assay was applied in 82.6% of genotoxicity studies with cytogenetic endpoints across various bivalve groups, including mussels, oysters, clams, cockles, and scallops, at different life stages. Compared to chromosomal structural and numerical aberration analysis, the micronucleus assay requires less specialized skills. This is particularly advantageous since chromosomal aberration assays cannot be used without prior knowledge of the diploid number (for numerical aberrations) and karyotype (for structural aberrations) of the tested species. However, chromosomal aberration analysis can provide additional information beyond what the micronucleus assay can offer, such as differentiating between clastogenic agents—which cause chromosome deletions, duplications, and translocation—and aneugenic agents—which cause aneuploidy. Applications of chromosomal structural and numerical aberration detection methodologies included various groups of bivalves but were geographically limited to Europe, with the exception of two studies recently performed in Qatar on the pearl oyster *Pinctada radiata* [15,16]. On the other hand, studies using the SCE assay were exclusively limited to mussels and ceased after Cornet’s publication [17]. The main concerns regarding this test remain unresolved, such as the preservation of intact labeled metaphases and the maintenance of a high-yield cell division rate for two or more cycles, both essential to ensuring the integrity and validity of the test [13,18]. Overall, research on bivalve genotoxicity utilizing cytogenetic endpoints continues to thrive, with a tendency to favor the micronucleus test over the others due to its simplicity and cost-effectiveness. Following is a brief summary of the main results of genotoxic studies for each cytogenetic endpoint technique, including recent methodological improvements.

#### 2.1.1. Chromosomal Aberration(s)

In 1982 [12], Dixon assessed genotoxic damage in *Mytilus edulis* from polluted harbor sites. Through the examination of embryos, he noted a significantly higher aneuploidy level in cells from samples from contaminated sites. Shortly after, the use of *Mytilus* spp. mussels in environmental monitoring investigations was extended to sites that are not typically inhabited by mussels as part of the Mussel Watch Program in non-occupied sites [19]. The Mussel Watch Program by the National Centers for Coastal Ocean Science (NCCOS) initiated coastal monitoring projects to investigate the toxicity of a wide range of emerging contaminants. At the forefront of investigated toxicants was the anti-biofouling agent tributyltin (TBT), which was commonly used in the 1970s and was only effectively banned in 2008 [20]. In 1986, Dixon and Prosser published their work on how different concentrations of TBT ranging from 0.05 μg/L to 1 μg/L could cause cytotoxic effects on *M. edulis* embryos, but they concluded that this agent did not induce aneuploidy. However, Jha et al. [21] provided evidence for TBT’s involvement in inducing aneuploidy in 12-h-old mussel embryos, the same age as those used by Dixon and Prosser [22]. This difference was attributed to the duration of embryo exposure to the stressor, during which cell cycle dynamics must be taken into account [21]. Jha et al.’s team from Plymouth Research Center produced three publications—including the one just above—on the effect of dredging on the coastal line and the effect of TBT and its derivative TBTO (tributyltin oxide) on *M. edulis* larvae [21,23,24]. There was a positive correlation between induced chromosomal damage and contaminant concentration [21,23] and time [24]. Studies of numerical chromosomal aberrations were not always restricted to the traditional method of direct chromosomal counting with the use of the air-drying technique; the use of flow cytometry was introduced to this field in 2003 by Bihari et al. [25]. In this study, cell cycle alterations were observed to measure the influence of poor environmental conditions on mussel health, demonstrating the possibility of using flow cytometry as a sensitive tool to spot changes in the DNA cell cycle profile and ploidy levels throughout the different phases of the cell cycle.

The first study assessing genotoxicity in oysters was conducted by Bouilly et al. [26], who investigated the effects of the pesticide atrazine on adults and juveniles of the Pacific oyster *Crassostrea gigas*. Through a controlled laboratory experiment, the authors confirmed the genotoxic effects of atrazine, which showed a clear dose-dependent relationship. Embryos and larvae of the same species were tested by Cheung et al. [27], and the aneuploidy level was found to increase with the concentration of the alkylating agent methyl methane sulfonate (MMS) or the endocrine disturber benzo[a]pyrene (B[a]P). However, the number of aneuploid metaphases decreased at the highest concentration of B[a]b. The authors suggested that the dose-dependent relationship between a genotoxic agent and its effect is limited by the cell’s ability to metabolize that agent; once the toxicity threshold has exceeded that limit, observations of cytogenetic aberrations decrease due to a low cell division rate. The utilization of complementary techniques such as flow cytometry to unravel the cell proliferation state would be an advantage in these studies.

The previously mentioned studies provided strong cytogenetic evidence of genotoxicity in *Mytilus* sp. and *C. gigas* across different life stages. However, the long-term toxic effects remained unclear, particularly the potential genotoxic consequences of parental exposure to toxic agent(s) on bivalve offspring. To address this knowledge gap, a new cohort of genotoxic studies emerged. In 2004, the persistence of aneuploidy levels across generations was demonstrated in bivalves, *C. gigas* oyster, in particular. Studies confirmed that juveniles—although not directly exposed to a genotoxic agent—could inherit genetic damage from a parent previously exposed to atrazine [28,29] or diuron [29,30]. Unlike alterations in hemocyte parameters, chromosomal damage induced by exposure to diuron was irreversible [29]. Chromosome loss due to aneuploidy might result in the absence of genetic regions, including crucial genes, which can lead to severe physiological consequences such as disrupted sexual maturity and reduced embryo survival rates [29,30]. To assess such genetic impacts, Barranger et al. [30] pioneered the use of fluorescent in situ hybridization (FISH) for conducting genotoxic assays in bivalves. The experiment examined the progeny of *C. gigas* parents exposed to diuron during gonadal development. Aneuploidy in embryos affected the stability of DNA regions containing the 5S and 18-5.8-28S rRNA genes on chromosomes 4, 5, and 10. Notably, the selected doses of atrazine in this study were environmentally relevant. More recently, persisting aneuploidy has also been observed in other bivalve species. In 2017, persistent aneuploidy in *Ruditapes philippinarum* clams from a site highly contaminated with metals was found to be strongly correlated with sediment contamination rather than seasonal variations [31]. The authors concluded that aneuploidy was primarily influenced by contaminants in sediment rather than being a direct consequence of temporal fluctuations in bioavailable contaminants. The authors also observed vertical transmission of aneuploidy in *R. philippinarum*, which was attributed to long-term exposure to metal contaminants in the sediment.

Another case of season-independent persistent aneuploidy was reported by Leitão et al. [15] in the pearl oyster *P. radiata* in the Arabian Gulf. Aneuploidy levels were primarily associated with specific contaminants—mainly mercury and polycyclic aromatic hydrocarbons (PAHs)—accumulated in the oyster tissues rather than being a direct response to the seasonal fluctuations in bioavailable contaminants. The authors suggested that this persistent aneuploidy likely reflects chronic exposure to site-specific contaminants rather than a direct consequence of bioavailability. This raised an important question on the longevity of the genotoxic effects of contaminants on bivalves. In an attempt to answer that question, a translocation experiment investigated the potential for recovery in oysters translocated to sites with significantly different chemical compositions [16]. The authors observed a pattern of aneuploidy reduction in translocated oysters compared to controls, regardless of the contaminant levels in their tissues, although the change was not statistically significant. This pattern suggests that sediment composition may play a crucial role in influencing aneuploidy recovery in bivalves.

Most studies employing chromosomal abnormalities as endpoints have predominantly focused on numerical rather than structural alterations. This preference stems from the fact that detecting structural abnormalities is more technically demanding, as it requires the establishment of an optimal karyotype at the chromatin condensation level and the possibility of applying differential chromosomal banding techniques. Notably, Cheung et al. [27] reported chromatid breakages in metaphase chromosomes of *C. gigas* exposed to MMS and B[a]P. In a related context, Leitão et al. [32] hypothesized that chromosomal fission events could be triggered by environmental stressors. Their study investigated *Cerastoderma edule* cockles from Galicia, a region with a documented history of oil spill exposure.

#### 2.1.2. Sister Chromatid Exchange (SCE)

SCE is a natural phenomenon in which the arms of chromosomes (sister chromatids) exchange genetic material to allow the genetic recombination and repair of genetic material/DNA damage. Arms labeled with bromodeoxyuridine (BrdU) can be traced in the following cell cycles, making observations of the exchange rate possible. Studies indicate that BrdU generates consistently low levels of SCE; thus, it has been used in control samples for labeling and as a genotoxic agent in some studies (e.g., [13,14]). Applications of the SCE test have focused on using embryos and larvae for two main reasons: the sensitivity of these developmental stages to chemical contamination and the brief experimental duration. The work of Dixon and Clarke [13] and Harrison and Johnes [14] unveiled a dose-dependent response relationship between BrdU, the alkylating agents mitomycin C (MMC) and MMS, and SCE in both the adults and larvae of *M. edulis*. Dixon and Prosser [22] tested the same species, and for the first time, both SCE and numerical chromosomal aberration assays were used together to measure the impact of TBTO and phenobarbital (PB) on larvae. In this study, TBTO did not cause cytogenetic damage, even in the presence of the known carcinogen phenobarbital (PB). On the other hand, in another study, the SCE level in *M. galloprovincialis* treated with carcinogenic mercury was two times higher than in those left untreated [33]. However, nitrilotriacetic acid—another known carcinogen for mammals—did not induce SCE and did not have synergetic effects on mercury genotoxicity [33]. A fact that cannot be overlooked is that the variability in results among SCE studies can be quite common due to several factors, including the time and duration of experimental exposure. Moreover, the random incorporation of the substance of interest without considering the cell cycle phase at the exposure time may produce misleading data [21,23]. Such information could be explored through in vitro tests of SCE, as demonstrated in the pilot study by Cornet [17], which involved BrdU incorporation into *M. galloprovincialis* mantle cell cultures. However, the data obtained were limited, and the methodological details were insufficient to ensure reproducibility, reflecting the broader challenges still faced in the field of bivalve cell culture.

#### 2.1.3. Micronucleus Induction (MN)

A common finding in several studies utilizing assays based on micronuclei and other nuclear abnormalities in bivalves is a peak in the micronucleus rate shortly after exposure, which then declines over a few days of exposure and stabilizes at a level approximately twice the control for weeks (e.g., [34,35]). Recovery to initial baseline levels requires a longer depuration time. For example, in a study by Machado-Schiaffino et al. [36], mussels recovered after approximately six months. Siu et al. [37] noted a delayed increase in micronucleus formation after low doses of B[a]P exposure over the course of four weeks. Jaeschke et al. [38] noted the recovery of the micronucleus frequency after 21 days of depuration in *M. edulis* exposed to tritiated water. On the other hand, Politakis et al. [39] reported that a shorter depuration period of just 7 days was sufficient for *M. galloprovincialis* hemocytes to return to baseline micronucleus levels following exposure to paracetamol, with values no longer significantly different from those of the control specimens. Unlike the acute response that they had observed at higher concentrations of the same contaminant, this increase in genotoxicity persisted, highlighting the prolonged effects of lower, chronic exposures. Falfushynska et al. [40] found persistent nuclear abnormalities that lasted 14 days following a low dose of radiation exposure of 2 mGy in *Anodonta anatina* mussels. The micronucleus frequency is influenced by several factors, including the mitotic division index of cells [41,42], the cell type involved [43], and even abiotic aspects such as temperature [44]. The intraindividual variation in micronucleus frequencies in one sample also remains a challenge. Several cellular mechanisms, such as lower division index (e.g., [34,41]), cell death [45], and new cell turnover [46,47], may help to prevent further micronucleus formation. While valve closure was thought to contribute to reduced genotoxicity, experiments on embryos supported the prior hypothesis that other mechanisms play a role in limiting micronucleus production [42].

An interesting point raised by Falfushynska et al. [48] was that the origin of the tested species had a major influence on genotoxic responses rather than exposure conditions alone. It was demonstrated in *A. anatina* mussels that even after 14 days of exposure to copper, zinc, and cadmium, nuclear abnormalities were still correlated with the genotoxic contaminant levels found at their site of origin. To minimize such confounding effects, it is recommended to either prolong accumulation and depuration periods in clean conditions before starting to test individuals in pristine conditions or acquire test organisms from well-characterized, uncontaminated reference sites whenever possible. Tissue-specific toxicity has been addressed several times in the literature, such as in the work of Butrimavičienė et al. [49], where gill cells responded faster than hemocytes after laboratory exposure to metals in *Anodonta cygnea*. It is likely that *Perumytilus purpuratus* gills were more sensitive than hemocytes after copper exposure [50]. These findings were attributed to the fact that the gill is in direct contact with the outer environment, in contrast to hemocytes [50].

In the standard protocol of the micronucleus assay, nuclear abnormalities are typically expressed per 1000 cells to ensure consistency and comparability across studies. Reporting values as percentages of total observed cells, as seen in Abdulla et al.’s study [51], where frequencies as high as 45.57% were noted, may lead to misinterpretation and reduced accuracy, as it deviates from established reporting conventions.

Several recommendations have been proposed to improve micronucleus identification and scoring systems, including the use of automated systems and the standardization of the number of tested individuals (e.g., [46,52,53]). However, the majority of subsequent studies continued to rely on the previous classical methods. Micronucleus formation has been applied as a genotoxicity assessment technique in a large number of monitoring surveys (e.g., [54,55,56]), experimental approaches (e.g., [43,57,58]), and translocation studies [59,60] involving multiple bivalve species. In the majority of these studies, a consistent positive correlation was observed between site-specific pollution levels and micronuclei formation, reinforcing the assay’s reliability and sensitivity as a bioindicator of environmental genotoxicity.

### 2.2. DNA Damage Endpoints

Efforts to understand the immediate genotoxic impact that causes DNA alterations or adjustment have driven the advancement of the following assays in bivalves: DNA polymerase activity, DNA unwinding and alkaline elution, DNA adducts, and the comet assay [61,62,63,64,65]. DNA-molecule-based assays are not dependent on the cell division rate as cytogenetic assays are, but they can be more logistically demanding. The first DNA-molecule-based study in bivalves [61] evaluated the rate of DNA repair in isolated digestive gland and gill cells from *M. galloprovincialis* exposed to dimethyl sulfate (DMS). The results showed a significant inhibition of DNA polymerase activity in the gill cells but not in the digestive gland. In a subsequent study, the same methodological approach was applied to test the genotoxicity of heavy metals in the digestive gland cells. DNA repair was significantly inhibited in treatments with high concentrations of metals [66]. In the DNA unwinding assay, the mechanism and timing of double-stranded DNA unwinding are analyzed, where the smaller the molecular weight, the shorter the time required for DNA to unravel. This assay was used to assess the genotoxic impacts of agents that cause DNA strand breakage; hence, any DNA strand breakage can be a starting point for the unwinding process. One of the shortcomings of this test was the lack of a real quantitative measurement that reflects the generated level of DNA breakage [67]. Moreover, as in many other assays, DNA unwinding is subjected to physical and chemical artifacts that might contribute significantly to DNA breaks [67,68].

**Figure 3:**
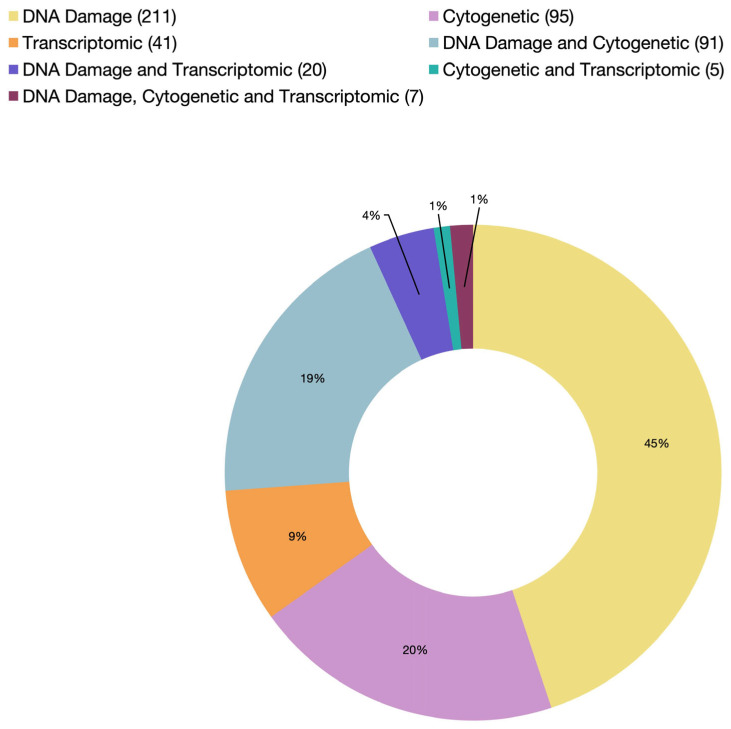
A summary of cited articles by endpoint category from 1982 to 2025 (the exact number of articles in each category is indicated in parentheses in the legend).

The alkaline single-cell gel electrophoresis/comet assay was first established in 1988 in an effort to develop an assay that measures damage and recovery in a single cell, quantifies DNA breakage in single and double strands, and eliminates RNA by alkalinization [68]. Consequently, since its first utilization in bivalve genotoxicity testing in 1997 by Sasaki et al. [65], the comet assay has become the most widely used method to assess DNA strand breakage in bivalves, accounting for 76% of such assays reported in the literature (Figure 3; Supplementary Table S2).

#### Alkaline Single-Cell Gel Electrophoresis/Comet Assay

The earliest publications that introduced the comet assay in bivalve genotoxicity testing were centered on seawater monitoring, both in controlled laboratory set-ups and in field experiments [65,69,70,71]. Some of these studies investigated the effects of mutagens such as 3-chloro-4-dichloromethyl-5-hydroxy-2(H)-furanone (MX) and B[a]P on bivalve models. However, the exposure methods varied between studies. Sasaki et al. [65] conducted in vivo exposure experiments on *Mizuhopecten yessoensis* scallops and *R. philippinarum* clams, while Mitchelmore et al. [69] performed an in vitro exposure test on isolated digestive gland cells from *M. edulis* mussels. Interestingly, the two studies used the same exposure time of 4 h, which was enough to induce detectable genotoxic effects. This observation raised the question of the timeframe required for DNA damage to be reversible across different species and whether the extent of the genotoxic damage had been assessed fairly. In vivo exposure involves the organism’s full physiological and innate immune system response, whereas the in vitro assays isolate specific tissues, potentially limiting the complexity of the biological response and influencing the observed outcomes. Wilson et al. [71] tackled this issue by applying the comet assay on *M. edulis* both in vivo and in vitro. Although the in vivo exposure was as long as 14 days and the in vitro exposure lasted for only 1 h, the damage caused by the latter was significantly greater. Thus, the authors advised that the in vitro application of the comet assay on this species could be more useful. However, many subsequent studies that employed both in vivo and in vitro approaches did not provide sufficient evidence favoring one approach over the other, largely due to differences in the experimental design, species-specific response, cell type, and genotoxic agent. Rigonato et al. [72] investigated the recovery of the freshwater clam *Corbicula fluminea* following exposure to MMS and observed that the clams recovered within 9 days post-exposure. This experiment underlined the importance of incorporating an acclimation period prior to conducting the comet assay—a point also emphasized by Rigonato et al. [73], who recommended a 30-day acclimation period for bivalves before experimentation with the comet assay. Aligning with that, Nagarajappa et al. [74] observed a decline in genotoxic effects in mussel gonads 12 days after tobacco exposure in an experimental setting where DNA damage was evaluated using the comet assay every 48 h for 16 days. These findings highlight a common limitation in many studies: the comet assay is performed at a single time point, which can overlook important temporal patterns in DNA damage and recovery. Including multiple time points allows for a more accurate assessment of genotoxicity and recovery dynamics.

Among the factors that must be considered when standardizing doses between in vivo and in vitro approaches, the cell type plays a particularly critical role. It was observed that the toxicity of biotoxins was cell-dependent in *R. decussatus*, with hemocytes and gill cells responding differently under in vivo and in vitro conditions [75]. Similarly, Prego-Faraldo et al. [76] reported a similar cell-type-specific sensitivity to okadaic acid toxicity in *M. galloprovincialis* under in vitro conditions. Interestingly, this pattern of cell-dependent response was not observed in a comparative in vivo study involving hemocytes and hepatopancreas cells of *M. galloprovincialis* and the Pacific oyster *C. gigas* following biotoxin exposure [77]. That being said, higher sensitivity to in vivo exposure compared to in vitro assays using hemocytes was documented in mussels by Gačić et al. [78]. This difference was attributed to the absence of hemocyte proliferation during the 22-h in vitro exposure period, potentially limiting the manifestation of genotoxic effects.

Together, these studies highlight the strengths and limitations of each approach: while in vitro assays provide controlled conditions for assessing cell-specific responses, in vivo systems can reveal complex organism-level interactions, such as immune responses, metabolic activity, and tissue connectivity, that can either amplify or mitigate toxicity outcomes. These insights reinforce the need to consider the cell type and physiological context not only in interpreting genotoxicity results but also when establishing dose equivalency between experimental approaches and experimental models. Failure to do so may lead to misleading comparisons between in vivo and in vitro data, with the risk of overlooking critical biological differences that influence toxicological responses. The type of genotoxic agent is another important variable when comparing in vivo and in vitro responses. For example, Pruski and Dixon [79] exposed *M. edulis* to cadmium chloride (CdCl_2_) both in vivo (0.2 mg/L) for 4 weeks and in vitro (0.7–15 mg/L) for 5 h, which resulted in nuclear damage and the disruption of DNA repair mechanisms. However, their findings did not clearly favor one exposure route over the other, and CdCl_2_ was considered only weakly genotoxic in their system. In contrast, Banakou et al. [80] reported a direct genotoxic effect of CdCl_2_ in *M. galloprovincialis* hemocytes under in vitro conditions at both lower and higher concentrations than those used by Pruski and Dixon [79]. Similarly, Slobodskova et al. [81] observed cadmium-induced DNA damage in *Corbicula japonica* following in vivo exposure. These differing results may reflect differences not only in species and cell types but also in how cadmium chloride interacts with cellular systems under isolated (in vitro) versus integrated physiological conditions (in vivo). Notably, cadmium has been used as a positive control in several comet assay protocols (e.g., [78,82]), further supporting its relevance as a genotoxic agent in both exposure modes. Together, these studies highlight how the nature of the genotoxic agent, along with biological and methodological factors, can significantly influence the comparability and interpretation of in vivo and in vitro data.

Several studies have suggested that the comet assay is more suitable for detecting genotoxicity in germ cells than in somatic cells due to the limited DNA repair mechanisms in the former [70,83]. For example, Lewis and Galloway [84] observed that three days after discontinuing the exposure of *M. edulis* to B[a]P, recovery from DNA damage in hemocytes was significantly higher compared to that in sperm cells, highlighting the differential repair capabilities between these cell types. In addition, the authors demonstrated that exposure of male *M. edulis* to B[a]P for three days induced genotoxicity in larvae without affecting fertilization rates. In contrast, a study by Kadar et al. [85] indicated that exposing *M. galloprovincialis* sperm to zero-valent nano-iron (nZVI) for just 2 h significantly reduced fertilization success and impaired embryonic development. Similar developmental disruptions were reported in *C. gigas* embryos following exposure to various metals [86]. These studies underscore the varying sensitivities of different cell types to genotoxic agents and suggest that sperm cells may be more vulnerable to certain contaminants, which could have long-term effects on reproductive success. Although, as mentioned earlier, over two-thirds of the published genotoxicity assays in bivalves utilized the comet assay, its adequacy as a standalone tool for assessing genotoxicity is still currently questionable. Bellas et al. [87] highlighted several limitations in its application, suggesting that it may not fully capture the complexity of genotoxic effects in environmental settings. In their study, *M. edulis* mussels were caged at a site undergoing dredging and significant sediment mobilization, which may have contributed to the observed limitations of the comet assay in detecting genotoxicity under these conditions.

### 2.3. Transcriptomic Endpoints

Since its emergence in 2002, two decades after the first genotoxicity studies on bivalves and in parallel with the rapid development of next-generation sequencing technologies, the application of transcriptome-level analysis to genotoxicity assessment has significantly increased (Figure 1). Such growing interest has manifested in investigating the effects of various environmental stressors on gene expression profiles and associated functions. To date, a wide range of stressors have been investigated, including petrochemicals [88,89,90,91,92,93,94], metals [95,96,97,98,99], pesticides [100,101,102], biotoxins [103,104,105,106,107,108,109,110], plastics [111,112,113,114], nanomaterials [115], and abiotic parameters such as acidification [114] and UV radiation [90,116], among others (Table 1). Transcriptome analysis extends beyond observations of phenotypic changes by incorporating molecular techniques such as restriction fragment length polymorphism (RFLP), DNA fingerprinting, and gene amplification. One of the most powerful tools in transcriptomic studies is the reverse transcription polymerase chain reaction (RT-PCR), which enables the detection and quantification of gene expression changes using specific primers targeting genes of interest. Rodius et al. [88] used RNA arbitrarily primed PCR (RAP-PCR) to test the impact of heavily contaminated sediment containing a mix of organic and inorganic pollutants on the freshwater mussel *Unio tumidus*. The study provided evidence of the suitability of using this technique in the genotoxicity assessment of bivalves. The authors found a PCR product that was only present in mussels exposed to contaminants. However, the authors highlighted a key limitation of RAP-PCR: its inability to clearly distinguish between DNA damage and differential gene expression. Rodius et al. [88] suggested that this limitation could be overcome by combining RAP-PCR with other complementary genotoxicity assays that detect DNA damage, such as the micronucleus or comet assay. Moreover, bivalves have also played a role in the evolution of cancer research and the genotoxicity testing of cancer treatments [117]. For instance, bioactive extracts from the clams *Donax variabilis*, *Donax incarnatus*, and *Donax cuneatus* and the mussel *Perna viridis,* which are rich in polysaccharides, have shown antiproliferative effects on human cancer cell lines, highlighting their potential as candidates for cancer drug testing or development [118].

The overall trends in cytogenetic, DNA damage, and transcriptomic assays in genotoxicity testing in bivalves show a shift from qualitative to quantitative methods, from manual scoring to automated analysis, and from single-tissue assessment to multiple-tissue investigations. A movement toward multi-assay integration for more comprehensive genotoxicity profiling is also noted. Additionally, efforts to standardize experimental protocols across laboratories have increased, ensuring better cross-study comparability and reproducibility. These advancements collectively reflect a broader move toward more reliable, objective, and mechanistically informed approaches in assessing genotoxicity in bivalves.

## 3. Discussion

### 3.1. The Use of Several Endpoints in a Single Study: Advantages and Key Findings

Integrating multiple genotoxicity assays within a single study provides a more comprehensive assessment of DNA damage at different biological levels and in different tissues. Among the various assay combinations, the micronucleus and comet assays are used together more frequently than any other tests (Figure 3). On the other hand, combining cytogenetic analysis (e.g., micronucleus or chromosomal aberration tests) and DNA-molecule-based analysis (e.g., comet assay) can help identify early, potentially repairable DNA damage caused by clastogenic agents before progressing to chromosomal alterations detectable at the cytogenetic level. Bolognesi et al. [163] were among the first to apply this integrative approach, combining at least two genotoxicity markers with distinct biological endpoints: the micronucleus assay (a cytogenetic endpoint) and alkaline elution (a DNA-molecule-base assay). The objective was to compare the sensitivity of these assays in detecting genotoxic effects in organisms exposed to heavy metals. Both assays successfully detected clastogenic effects induced by metals such as copper and mercury. However, discrepancies arose as to whether the observed DNA damage exhibited a clear dose–response relationship or a direct cause–effect interaction. Within the same year, Bresler et al. [164] monitored the health of several marine species along the Red Sea and Mediterranean coasts using a battery of genotoxicity biomarkers, including the micronucleus assay and DNA unwinding assay. A positive correlation was found between genotoxic effects and site-specific contamination levels. This pattern was particularly evident in the clam *Donax trunculus,* which exhibited greater sensitivity compared to other tested models, such as the clam *Cyrenoida floridana* and the mussel *M. edulis*.

A key distinction between cytogenetic tests and DNA-molecule-based assays lies in the persistence and detectability of DNA damage. For instance, the comet assay detects transient DNA damage that may be repaired over time, making it particularly useful for assessing short-term genotoxic effects and DNA repair mechanisms. In contrast, cytogenetic assessments are more suitable for detecting long-term, cumulative genotoxic effects, making them more appropriate for controlled laboratory experiments involving longer exposure durations.

While the genotoxic effects of chemical agents are well documented at the chromosomal and DNA strand levels, transcriptomic responses only started to be investigated in 2007, when studies started integrating gene expression analysis with traditional genotoxic endpoints. For instance, Di et al. [165] investigated the effects of B[a]P exposure in *M. edulis* using both the comet assay and transcriptomic analysis of the tumor-regulating genes p53 and ras. The study demonstrated that B[a]P exposure not only caused significant DNA strand breaks but also upregulated the expression of p53 and ras genes in hemocytes [133]. Another well-known genotoxic agent is mercury, which has been shown to cause chromosomal aberrations, DNA strand breaks, and micronuclei, among other effects, in humans [166,167,168], as well as in aquatic organisms such as *Andinoacara rivulatus* fish assessed with the micronucleus test [169] and *Palaemon khori* shrimp tested using aneuploidy assessment [170]. Yet, until recently, little was known regarding its molecular toxicity pathways. Pytharopoulou et al. [142] provided novel insights into mercury toxicity by finding its impact on the 40s ribosomal subunit, resulting in disturbed protein synthesis in mussels and contributing to micronucleus formation. Since then, studies combining multiple genotoxicity endpoints, including cytogenetic, molecular, and transcriptomic approaches, have expanded to investigate the effects of a wide range of chemical and physical stressors. The first two experimental studies integrating all three major genotoxicity assessment endpoints—cytogenetic, DNA strand break, and transcriptomic analysis—focused on the effects of nanoparticles and microplastics on *M. galloprovincialis* [144,148]. Canesi et al. [148] found that titanium oxide (TiO_2_) nanoparticles induced less genotoxicity than titanium oxide’s derivative 2,3,7,8-tetrachlorodibenzo-p-dioxins (2,3,7,8-TCDD), which is commonly known to be the most toxic form of TiO_2_. The three endpoints of the study showed evidence that TCCD is more toxic both in vivo and in vitro. In Avio et al.’s [144] study, a comprehensive battery of biomarkers, including DNA microarrays, micronuclei, and the comet assay, were utilized to assess the impact of microplastics. It was observed that pyrene-loaded polystyrene and polyethylene particles led to a higher micronucleus frequency in comparison to virgin microplastics. Interestingly, the comet assay showed the opposite pattern: mussels exposed to virgin microplastics exhibited higher levels of DNA breakage. The authors attributed this intriguing finding to the faster detectability of DNA strand breaks relative to micronucleus formation. Under more severe genotoxic stress, such as exposure to pyrene-loaded particles, the extent of DNA damage intensifies to the point of causing nuclear deformation, thereby increasing the frequency of micronuclei. Furthermore, transcriptomic analysis revealed both the upregulation and downregulation of various genes across treatments. Many of those genes are involved in vital molecular pathways, including detoxification, oxidative stress, immune responses, and cell cycle regulation.

Collectively, these findings highlight that the use of multiple genotoxicity endpoints, each targeting different biological levels, not only enhances detection sensitivity but also provides a comprehensive, multidimensional view of toxicological effects, thereby supporting more robust environmental risk assessments. For instance, the comet assay is particularly effective for detecting reversible DNA strand breaks and assessing DNA repair mechanisms, while the micronucleus assay detects more stable chromosomal alterations. Complementing these, transcriptomic analysis offers insights into gene expression pattern changes triggered by specific stressors, offering insights into affected molecular pathways. By integrating these complementary approaches, both transient and cumulative genotoxic effects can be detected—effects that might otherwise be missed when relying on a single assay.

### 3.2. Challenges in Genotoxicity Testing

As more efforts are being put into investigating the risks associated with exposure to genotoxic compounds, the number of published studies on this topic has increased significantly in the last four decades. Despite this progress, several challenges still persist in effectively utilizing existing knowledge, developing reliable testing assays, and drawing consistent, clear conclusions. One major difficulty in genotoxicity testing is the lack of standardized global guidelines for assay selection, data interpretation, and result validation. Some of these difficulties have been mitigated by the development of search engines, searchable databases, and data-sharing platforms, facilitating access to information and enhancing collaboration among researchers worldwide. However, the absence of universally standardized protocols continues to limit the comparability and reproducibility of the studies. Standardized guidelines are crucial for ensuring the selection of appropriate genotoxic approaches and bioassays, achieving statistically robust results, and providing realistic measures of genotoxic risk assessment. Addressing these elements comprehensively is essential for improving the consistency and reliability of genotoxicity testing. In 2015, in response to these challenges, the Organization for Economic Co-operation and Development (OECD) established the Genetic Toxicology Test Guidelines (TGs). These guidelines address many of the previous issues and aim to standardize genotoxicity testing by providing clear procedures for conducting assays across different study designs and laboratory settings. By establishing standardized methodologies, the OECD TGs enhance the consistency, reliability, and global comparability of genotoxicity assessments while also helping to reduce the variability in test outcomes and promote best practices in biological testing worldwide.

## 4. Concluding Remarks, Prospects, and Recommendations

Genotoxicity research in bivalves has made, as presented, considerable progress over recent decades, driven by growing environmental concerns and advances in molecular/omics tools. However, key methodological gaps highlight the need for more standardized, informed, and integrative approaches moving forward. To support the continued development and application of genotoxicity tools in bivalves, we recommend the following work areas to enhance their effectiveness.

The availability of genotoxic data in online public databases would increase the impact of existing research and pave the way for further advancements. This would improve the reproducibility of experimental work and support the integration of regional frameworks into more global applications of genotoxicity testing. We propose transforming the sources cited in this review and presented in the Supplementary Material into an open-access online database that can be continuously updated with future studies. This centralized resource would reduce duplicated efforts and offer inclusive support to researchers, policymakers, and other stakeholders. By making the data openly accessible, we aim to foster transparency, facilitate collaboration, and encourage broader engagement across the genotoxicity research community.

The lack of standardized methodologies remains a central challenge in genotoxicity testing. With the increasing number of genotoxicity studies, there is also growing recognition of diverse bivalve species as model organisms and an expanding variety of contaminants being investigated. However, despite this progress, there is still no consensus on quality assurance protocols, and quality control measures are inconsistently applied. Several experimental results are often influenced by the prior exposure history of collected organisms. Acclimation periods prior to experimentation are essential to mitigate this influence; however, their optimal duration can vary significantly depending on both the species and the type of assay used. Furthermore, it is now understood that genotoxic effects may be transmitted vertically across generations, reinforcing the need for more in-depth research into the persistence of DNA damage over time.

The standardization of bivalve genotoxicity testing could be advanced through the development of long-term cell lines from representative model species. However, this area of research remains underdeveloped in invertebrates due to the technical challenges associated with culturing and maintaining invertebrate cells over extended periods. While a few successful primary cell cultures have been established—such as in *M. edulis* for up to 22 months [171] and in *Crassostrea madrasensis* for 1 month [172]—their integration into genotoxicity testing frameworks has yet to be realized.

Assays such as SCE, chromosomal aberrations, and the micronucleus test are being refined and gaining recognition as reliable genotoxicity indicators. In vitro testing approaches also began to be incorporated into genotoxicity assessment frameworks during that period. In the late 1990s and early 2000s, advancements in genomics and molecular genetics ushered in a new era for genotoxicity testing. Techniques such as gene expression profiling emerged as powerful tools, enabling more mechanistic and predictive evaluations of genotoxic responses. Applying comprehensive omics tools—such as transcriptomics, proteomics, and metabolomics—holds great potential for providing deeper insights into the molecular pathways affected by genotoxins and can reveal tissue- or cell-specific sensitivities that traditional methods may overlook. However, despite the increasing use of transcriptomics in bivalve genotoxicity research—reported in at least 74 studies—only three have employed an in vitro approach. This highlights a critical gap and opportunity: integrating omics technologies with in vitro systems could yield more controlled, mechanistically insightful, and reproducible outcomes, significantly advancing the field.

When applying any of the aforementioned genotoxicity assays in environmental monitoring, it is essential to consider the nature of the contaminants and their associated molecular pathways. These assays typically reflect the cumulative impact of all environmental stressors, without identifying the specific agents responsible for the observed effects. As such, results must be interpreted with caution and within the context of known or suspected exposure scenarios. The genotoxic responses observed may be the outcome of complex interactions among multiple contaminants, as well as abiotic factors such as temperature, salinity, and pH. These interactions can either amplify or mitigate genotoxic effects, complicating the attribution of damage to a single causative agent. Therefore, a comprehensive understanding of the environmental context, including chemical analyses and ecological parameters, is critical for drawing meaningful conclusions from genotoxicity data.

The application of the above recommendations would be a step forward in the understanding of genotoxic impacts in bivalves, enabling more accurate risk assessments, improved environmental monitoring, and the development of globally relevant and aligned testing strategies.

## Figures and Tables

**Table 1:** Published studies with transcriptomic endpoints since 2002.

No.	Species	Stressor	End Point	Investigation Type	Exposure Type	Exposure Duration	Concurrent Endpoints	Reference
1	*Chlamys farreri*	Benzo[a]Pyrene (B[a]P)	Expression of genes related to detoxification, oxidative stress, and metabolic processes	Laboratory	in-vivo	10 days		[92]
2	*Chlamys farreri*	Benzo[a]Pyrene (B[a]P)	Expression of genes of genes related to ovarian development: collagen alpha-3VI (COL6A3), integrin alpha-9 (ITGA9), 17⍺hydroxylase-17,20-lyase (CYP17), 17β-hydroxysteroid dehydrogenases (17β-HSD), 3β-hydroxysteroid dehydrogenases (3β-HSD), estrogen receptor (ER), vitallogenin (VTG) and house keeping gene (β-actin).	Laboratory	in-vivo	10 days		[94]
3	*Chlamys farreri*	Estrogen 7*b-estradiol (E2)*	Expression of genes of genes related to reproduction, endocrine regulation, and metabolism	Laboratory	in-vivo	10 days		[119]
4	*Corbicula fluminea*	Benzotriazole UV stabilizer-329 (UV-329)	Expression of genes with focus on antioxidants and caspases	Laboratory	in-vivo	21 days		[120]
5	*Corbicula fluminea*	Nanoplastics, Microplastics	Expression of genes related to cellular components and apoptosis	Laboratory	in-vivo	10 days		[112]
6	*Crassostrea brasiliana*	Water-Accommodated Fraction (WAF)	Expression of genes involved in protein regulation, immune and stress response	Laboratory	in-vivo	1 day		[121]
7	*Crassostrea gigas*	Atrazine	Expression of genes related to enzymatic activities: superoxide dismutase (SOD), Catalase (CAT), heat shock protein (HSP), glutathione (GSH), glutathione-S-transferase (GSTs), Na+/K+-ATPase and acetylcholinesterase (AChE).	Laboratory	in-vivo	7 days		[102]
8	*Crassostrea gigas*	Benzo[a]Pyrene (B[a]P), Estrogenic 17⍺-ethinylestradiol (EE2), Endosulfan (ES)	DNA oxidation through measuring 8-oxodGuo	Laboratory	in-vivo	16 h	Comet assay	[122]
9	*Crassostrea gigas*	Biotoxin (Gymnodinium catenatum)	Expression of genes involved in cell cycle regulation (p21, p53, cafp55) and initial inflammatory (caspase 1 (casp1))	Laboratory	in-vivo	1, 7 and 14 days		[106]
10	*Crassostrea gigas*	Biotoxin (Prorocentrum lima, Karenia brevis) and bacteria (Vibrio parahaemolyticus, V. campbellii., V. parahaemolyticus)	Expression of genes related to apoptotic caspases 2, 3, 7, and 8.	Laboratory	in-vitro	4-6 h	DNA fragmentation, chromatin density, comet assay	[123]
11	*Crassostrea gigas*	Biotoxin (Prorocentrum lima)	Expression of genes related to cell cycle regulator and immune response (Cg-p21, Cg-CAFp55, Cg-EF2, β-1 and Cg-LGBP)	Laboratory	in-vivo	0, 3, 6, 24, 72, 168 and 336 h		[105]
12	*Crassostrea gigas*	Copper	Expression of genes related to homeotic, biomineralization and DNA methylation	Laboratory	in-vivo	3, 7 and 24 h	Comet assay	[124]
13	*Crassostrea gigas*	Diuron	Expression of genes related to oxidative stress and mitochondrial damage	Laboratory	in-vivo	2 periods, each is 7 days		[101]
14	*Crassostrea gigas*	Diuron	Expression of geness in genes involved in Stress response, Xenobiotic biodegradation, Antioxidative response, Apoptosis, DNA methylation, Gene transcription regulation, DNA recombination, DNA repair, DNA replication, DNA transcription and Cytokinesis	Laboratory	in-vivo	14 days		[100]
15	*Crassostrea gigas*	Diuron	DNA methylation	Laboratory	in-vivo	2 periods, each is 7 days	DNA adducts	[125]
16	*Crassostrea gigas*	Lead (Pb)	Expression of genes of genes (DEGs) related to endoplasmic reticulum (ER) stress and fatty acid oxidation	Laboratory	in-vivo	9 days		[98]
17	*Crassostrea gigas*	Metolachlor	Expression of genes involved in oxidative stress responses (mitochondrial superoxide dismutase and catalase: superoxide dismutase (sodmt), catalase (cat), glutathion peroxidase (gpx), metallothionein (mt1 & mt2), cytochrome C oxidase (cox1), cytochrome p450 (cyp1A), glutathion S-transferase (gst), multixenobiotic resistance gene (mxr), mitochondrial 12S ribosomal transcript (12S), tumor supressor (p53), house keeping gene (β-actin))	Laboratory	in-vivo	1 day	Comet assay	[126]
18	*Crassostrea gigas*	Pesticide, Copper	Transcription of genes involved in anti-oxidative stress (cat), respiratory chain (coxI), metal detoxification (mt1 and mt2), and cell cycle arrest and apoptosis (p53)	Laboratory	in-vivo	1 day	Comet assay	[127]
19	*Crassostrea gigas*	Radionuclides	Expression of genes related to stress	Laboratory	in-vivo	6, 11 and 14 days	Comet assay	[128]
20	*Crassostrea hongkongensis*	Metals	Expression of genes and proteomics (isobaric tags for relative and absolute quantification (iTRAQ))	Monitoring	-	-		[99]
21	*Crassostrea Virginia*	Biotoxin (Karenia brevis)	Expression of histone genes (H2A.X, H2A.Z, MacroH2A, GAPDH and RPL13) and global DNA mythelation	Laboratory	in-vivo	35 days		[107]
22	*Dreissena polymorpha*	Gadolinium	Expression profile of superoxide dismutase (SOD), catalase (CAT), metallothionein (MT), glutathione-S-transferase (GST), cytochrome c oxidase (CO1), cyclin D (Cyc D), lipid peroxidation (LPO), prostaglandin cyclooxygenase (COX).	Laboratory	in-vivo	28 days	Alkaline precepitation	[96]
23	*Dreissena polymorpha*	Samarium (Sm) and Yttrium (Y)	Expression profile of superoxide dismutase (SOD), catalase (CAT), metallothionein (MT), glutathione-S-transferase (GST), cytochrome c oxidase (CO1), cyclin D (Cyc D), lipid peroxidation (LPO), prostaglandin cyclooxygenase (COX).	Laboratory	in-vivo	28 days	Alkaline precepitation	[129]
24	*Dreissena polymorpha*	Unspecified (Monitoring Seine River)	Expression of genes involved in detoxification system and xenobiotic exposure	in-situ	Transplant	3 months	DNA adducts	[130]
25	*Dreissena polymorpha*, *Mytilus galloprovincialis*	Phosphorus P32, copper	Transcription of genes related to stress	Laboratory	in-vivo	10 days	Micronuclei, Comet assay	[131]
26	*Hybrid of M. edulis & M. trossulus*	UV filters	Expression of genes related to: oxidative stress (glutathione reductase), cellular stress response (cathepsin D), xenobiotic biotransformation system capacity (NADPH-cytochrome P450-oxidoreductase (CYP450 1A), monooxygenase, carboxylesterase and glutathione S-transferase), apoptosis (caspases 2 and 3, B-cell lymphoma (Bcl-2) and Bcl-2-associated X protein (BAX)), inflammation (nuclear factor κB(NF-κB) and interleukin IL-17), tumor supressor (p53), growth arrest and DNA-damage-inducible protein (GADD45), and lipid metabolism ((acetyl-CoA carboxylase (ACC), peroxisome proliferator-activated receptor (PPARγ) and cyclooxygenase 2 (COX-2)).	Laboratory	in-vivo	14 days		[116]
27	*Meretrix meretrix*	Nanoplastics	Expression of genes related to energy homeostasis and immunomodulation	Laboratory	in-vivo	7 days		[113]
28	*Mya arenaria*	Leptomycin B	Expression of tumor regulator gene (p53)	Laboratory	in-vivo	4, 8, and 24 h	Comet assay	[117]
29	*Mytilus chilensis*	Saxitoxin	Expression of genes involved in thermal stress, oxidative stress, metal contamination and pathogen response (heat shock proteins (HSP70 and HSP90), catalase (CAT), and superoxide dismutase (SOD), ferritin (Fer), metallothionein (Met), mytilin B (MytB), myticin A (MytA), pattern-recognition receptors (PGRP), fibrogen (Fib), transcription factor involved in the activation of the TNF-*α* gene (LITAF), galectin (Gal) and ependymin (Epe))	Laboratory	in-vivo	4, 6 and 48 h		[103]
30	*Mytilus coruscus*	Copper	Expression of genes related to environmental stress (metal ion binding, heat shock response and complement system)	Laboratory	in-vivo	18 days	Comet assay	[132]
31	*Mytilus coruscus*	Ocean acidification, Microplastics	Sequencing of gene 16S RNA	Laboratory	in-vivo	21 days		[114]
32	*Mytilus edulis*	Benzo[a]Pyrene (B[a]P)	Expression of genes involved in tumor regulation (p53 and ras)	Laboratory	in-vivo	6 and 12 days	Comet assay	[133]
33	*Mytilus edulis*	Heavy fuel oil, Styrene	Expression of genes related to cell cycle arrest and DNA repair (p53 and gadd45a)	Laboratory	in-vivo	3, 19 days and 5 months		[134]
34	*Mytilus edulis*	Trinitrotoluene (TNT)	Expression of carbonyl reductase (CR)	in-situ and Laboratory	Field exposure and in-vivo	4 days and 21 days in vivo, 58 days in Field experiment		[135]
35	*Mytilus galloprovincialis*	surfactants sodium dodecylbenzene sulfonate (SDBS) and sodium dodecyl sulfate (SDS)	Amplified fragment length polymorphism (AFLP)	Laboratory	in-vivo	72 days		[136]
36	*Mytilus galloprovincialis*	Benzo[a]Pyrene (B[a]P), C60 Fullerene	Transcriptional alterations of p53 and ras	Laboratory	in-vivo	3 days	Comet assay	[137]
37	*Mytilus galloprovincialis*	Benzo[a]Pyrene (B[a]P), Multiwalled carbon nanotubes (MWCNTs)	Global gene expression	Laboratory	in-vivo	3 days	Micronuclei, Comet assay	[138]
38	*Mytilus galloprovincialis*	Biotoxin (Prorocentrum lima)	Expression of genes involved in chromatin-associated and maintenance of genome integrity	Laboratory	in-vivo	1 day		[104]
39	*Mytilus galloprovincialis*	Biotoxin (Prorocentrum lima)	Expression of antioxidant genes: catalase (CAT), superoxide dismutase (SOD), Glutathione S-Transferase pi-1 (GST-pi) and Selenium-dependentGlutathione PeroXidase (Se-GPx). Also, Histone H2A and 18S rRNA	Laboratory	in-vivo	1 & 2 days		[108]
40	*Mytilus galloprovincialis*	Cadmium (Cadmium Telluride)	Metallothioneins (mt10IIIa and mt20IV)	Laboratory	in-vivo	14 days		[97]
41	*Mytilus galloprovincialis*	Copper	Expression of genes related to stress	Laboratory	Embryos	1 day	DNA plasmid	[139]
42	*Mytilus galloprovincialis*	Hydrogenated cement particle (HCP)	Expression of genes related to stress	Laboratory	in-vivo	16 days	Comet assay	[140]
43	*Mytilus galloprovincialis*	Mercury	Expression of genes related to stress	Laboratory	in-vivo	1 day	DNA fragmentation	[141]
44	*Mytilus galloprovincialis*	Mercury ions (Hg^2+^)	Expression of genes involved in protein synthesis	Laboratory	in-vivo	5, 10 and 15 days	Micronuclei	[142]
45	*Mytilus galloprovincialis*	Metals, temperature	Transcription of genes related to DNA repair and DNA replication	Laboratory	Embryos	2 days	Comet assay	[143]
46	*Mytilus galloprovincialis*	Microplastics	DNA microaarays of genes involved in immunological responses, lysosomal compartment, peroxisomal proliferation, antioxidant system and neurotoxic effects	Laboratory	in-vivo	7 days	Micronuclei, Comet assay	[144]
47	*Mytilus galloprovincialis*	Microplastics, Benzo[a]Pyrene (B[a]P)	Expression of genes related to DNA repair	Laboratory	in-vivo	1 & 3 days	Micronuclei	[145]
48	*Mytilus galloprovincialis*	Nano Plastics, Carbamazepine	Expression of genes related to DNA repair and biotransformation	Laboratory	in-vivo	2 days	Comet assay	[146]
49	*Mytilus galloprovincialis*	Nanoparticles (Ag)	Transcription of genes related to antioxidation response, detoxification response, stress, protein damage, apoptosis, Cellular death, houskeeping genes and xenobiotic metabolism	Laboratory	in-vivo	15 days		[115]
50	*Mytilus galloprovincialis*	Nanoparticles of Copper oxide (CuO NP)	Expression of genes related to DNA damage and cancer (ras, p53, and gadd45α)	Laboratory	in-vivo	21 days	Micronuclei	[147]
51	*Mytilus galloprovincialis*	Nanoparticles of Titanium Dioxide (n-TiO_2_) and 2,3,7,8-tetrachlorodibenzo-p-dioxins (2,3,7,8-TCDD)	Expression of genes related to antioxidant defence, stress response, apoptosis, tumor supressor and reproduction (glutathion S-transferase (GST), catalase (cat), heat shock protein (HSP70), (p53) and Estrogen Receptor genes (MeERs))	Laboratory	in-vitro, in-vivo	30 and 60 min in vitro or 4 days in-vivo	Micronuclei, Comet assay	[148]
52	*Mytilus galloprovincialis*	Nanoparticles of Titanium oxide and cadmium chloride (TiO_2_ NP, CdCl_2_)	Expression of genes involved in detoxification (ABC transporter)	Laboratory	in-vitro, in-vivo	2 h in-vitro or 4 days in-vivo	Comet assay	[149]
53	*Mytilus galloprovincialis*	NSAID (diclofenac, Ibuprofen & Ketoprofen)	Expression of genes involved in endocytosis, oxidation reduction, apoptosis, RNA processing, macromolecule catabolic process, NOD-like receptor signaling pathway, fatty acid metabolic and biosynthetic process, and Toll-like receptor signaling pathway	Laboratory	in-vivo	14, 30 & 60 days	Micronuclei, Comet assay	[150]
54	*Mytilus galloprovincialis*	Oil spill	Microsatellites (Mgu1, Mgu2, Mgu3, Mgu4, Mgu5, Mgu6, Mgu7)	Monitoring	-	-		[151]
55	*Mytilus galloprovincialis*	Oil, waste water	Expression of genes related to environmental pollution and hypoxia	Laboratory	in-vivo	15 days	Micronuclei, Comet assay	[152]
56	*Mytilus galloprovincialis*	PAHs, PCBs, UV	Expression of genes related apoptosis (caspase genes)	Laboratory	in-vitro	1, 3, 6 and 24 h		[90]
57	*Mytilus galloprovincialis*	pH, carbamazepine	Expression of genes related to immune responses, cellular homeostasis and oxidative system	Laboratory	in-vivo	28 days	Micronuclei, DNA fragmentation	[153]
58	*Mytilus galloprovincialis*	Tritiated water (HTO), temperature	Expression of genes related to Metal binding, protein folding, cell cycle chckpoint control and DNA repair	Laboratory	in-vivo	12, 72 and 168 h	Comet assay	[154]
59	*Mytilus galloprovincialis*	Water-Accommodated Fraction (WAF)	Expression of tumor regulator gene (ras)	Laboratory	in-vivo	1 day		[89]
60	*Mytilus galloprovincialis*	Zinc Pyrithione (ZnPT)	Expression of genes related to stress	Laboratory	in-vivo	14 days	Micronuclei, Comet assay	[155]
61	*Mytilus* spp.	Polystyrene Microplastics, Fluoranthene	Expression of genes involved in antioxidant enzymes activities: superoxide dismutase (SOD), catalase (CAT), Se-dependant-Glutathione peroxidise (gpx), Cytochrome P450 (cyp11 and cyp32), ***ω***-glutathione-s-transferase (***ω***gst), **μ**-glutathione-s-transferase (**μ**gst), **σ**-glutathione-s-transferase (**σ**gst), growth arrest and DNA damage inducible (aadd45a), a-amylase (amylase), pyruvate kinase (pk), Isocitrate dehydrogenase [NADP] cytoplasmic (idp), Gyceraldehyde 3 phosphate dehydrogenase (gapdh), hexokinase (hk), tumor supressor (p53), ABCB/P-glycoprotein-like protein (pgp), lysosome (lys), caspase 3/7-3 (casp37-3).	Laboratory	in-vivo	7 days		[111]
62	*Mytilus, Crassostrea gigas*	Benzo[a]Pyrene (B[a]P)	Expression of genes related to stress	Laboratory	in-vivo	3 days	Comet assay, DNA adducts	[156]
63	*Pecten maximus*	Biotoxin (domoic acid)	Expression of genes involved in vesicle-mediated transport, stress, signal transduction, immune system process, RNA metabolic process and autophagy	Laboratory	in-vivo	12 days		[110]
64	*Perna canaliculus*	Copper, Benzo[a]Pyrene (B[a]P)	Expression of genes involved in oxidative stress, xenobiotic transfer, membrane transportation, cellular and DNA response/repair, and endocrine disruption	Laboratory	in-vivo	2 days		[157]
65	*Perna viridis*	Benzo[a]Pyrene (B[a]P)	Expression of genes genes (DEGs) related to stress response, infectious disease and innate immunity	Laboratory	Embryos	1 day		[93]
66	*Perna viridis*	Biotoxin (Prorocentrum lima)	Transcription of genes involved in cytoskeleton, apoptosis, complement system and immune stress	Laboratory	in-vivo	4 days		[109]
67	*Ruditapes philippinarum*	Metals (cadmmium, mercury and lead)	Expression alterations of genes related to DNA damage and metal exposure (cytochrome C oxidase (cox1), cytochrome (cytb), superoxide dismutase (sod), catalase (cat) and 16S RNA)	Laboratory	in-vivo	8 days		[95]
68	*Ruditapes philippinarum*	Nanoplastics	Expression of geness in genes involved in: digestion, autophagy, and mitochondrial function and respiratory	Laboratory	in-vivo	35 days		[158]
69	*Ruditapes philippinarum*	Unspecified (Monitoring River Po, Italy)	Expression of genes related to oxidative and general stress responses, neuroendocrine response and, xenobiotic biotransformation	in-situ	Transplant	3 months	DNA adducts	[159]
70	*Tegillarca granosa*	Di-octyl Phthalate	Expression of genes related to immune response	Laboratory	in-vivo	7 and 14 days		[160]
71	*Tegillarca granosa*	Nanoparticles	Expression of genes related to metabolism	Laboratory	in-vivo	7 days		[161]
72	*Unio tumidus*	Polycyclic aromatic hydrocarbons (PAHs), Polychlorobiphenyls (PCBs) and Metals	Alterations in RNA arbitrarily primed polymerase chain reaction (RAP-PCR)	in-situ	Transplant	14 days		[88]
73	*Unio tumidus*	Unspecified (Monitoring Moselle River, France)	Genes involved in detoxification and antioxidation (Superoxide Dismutase (SOD), catalase (CAT), Selenium-dependent Glutathione Peroxidase (Se-GPx), Pi Class Glutathione S-Transferase (Pi-GST), and Metallothionein (MT))	in-situ	Transplant	8 and 21 days		[162]
74	*Venerupis philippinarum*	Benzo[a]Pyrene (B[a]P)	Expression of genes of genes involved in immune response (cathespin L2 cysteine protease, cathespin D, defesin, serine protease, thioester, scavennger receptor cysteine rich protein, C1q and 18S rRNA)	Laboratory	in-vivo	10 days		[91]

## Data Availability

Not applicable.

## Supplementary Materials

The following supporting information can be downloaded at https://www.mdpi.com/article/10.3390/ijms26115389/s1: References [22,37,48,54,163,164,173,174,175,176,177,178,179,180,181,182,183,184,185,186,187,188,189,190,191,192,193,194,195,196,197,198,199,200,201,202,203,204,205,206,207,208,209,210,211,212,213,214,215,216,217,218,219,220,221,222,223,224,225,226,227,228,229,230,231,232,233,234,235,236,237,238,239,240,241,242,243,244,245,246,247,248,249,250,251,252,253,254,255,256,257,258,259,260,261,262,263,264,265,266,267,268,269,270,271,272,273,274,275,276,277,278,279,280,281,282,283,284,285,286,287,288,289,290,291,292,293,294,295,296,297,298,299,300,301,302,303,304,305,306,307,308,309,310,311,312,313,314,315,316,317,318,319,320,321,322,323,324,325,326,327,328,329,330,331,332,333,334,335,336,337,338,339,340,341,342,343,344,345,346,347,348,349,350,351,352,353,354,355,356,357,358,359,360,361,362,363,364,365,366,367,368,369,370,371,372,373,374,375,376,377,378,379,380,381,382,383,384,385,386,387,388,389,390,391,392,393,394,395,396,397,398,399,400,401,402,403,404,405,406,407,408,409,410,411,412,413,414,415,416,417,418,419,420,421,422,423,424,425,426,427,428,429,430,431,432,433,434,435,436,437,438,439,440,441,442,443,444,445,446,447,448,449,450,451,452,453,454,455,456,457,458,459,460,461,462,463,464,465,466,467,468,469,470,471,472,473,474,475,476,477,478,479,480,481,482,483,484,485,486,487,488,489] are cited in the supplementary materials.
